# Can We Use 2,3,5-Triphenyltetrazolium Chloride-Stained Brain Slices for Other Purposes? The Application of Western Blotting

**DOI:** 10.3389/fnmol.2019.00181

**Published:** 2019-07-30

**Authors:** Sonia Sanchez-Bezanilla, Michael Nilsson, Frederick R. Walker, Lin Kooi Ong

**Affiliations:** ^1^School of Biomedical Sciences and Pharmacy and Priority Research Centre for Stroke and Brain Injury, The University of Newcastle, Callaghan, NSW, Australia; ^2^Hunter Medical Research Institute, New Lambton Heights, NSW, Australia; ^3^NHMRC Centre of Research Excellence in Stroke Rehabilitation and Brain Recovery, Heidelberg, VIC, Australia; ^4^Centre for Rehab Innovations, The University of Newcastle, Callaghan, NSW, Australia; ^5^Lee Kong Chian School of Medicine, Nanyang Technological University, Singapore, Singapore; ^6^School of Pharmacy, Monash University Malaysia, Bandar Sunway, Malaysia

**Keywords:** stroke, 2, 3, 5-triphenyltetrazolium chloride, western blotting, infarct, thalamus, hippocampus

## Abstract

2,3,5-Triphenyltetrazolium chloride (TTC) staining is a commonly used method to determine the volume of the cerebral infarction in experimental stroke models. The TTC staining protocol is considered to interfere with downstream analyses, and it is unclear whether TTC-stained brain samples can be used for biochemistry analyses. However, there is evidence indicating that, with proper optimization and handling, TTC-stained brains may remain viable for protein analyses. In the present study, we aimed to rigorously assess whether TTC can reliably be used for western blotting of various markers. In this study, brain samples obtained from C57BL/6 male mice were treated with TTC (TTC+) or left untreated (TTC−) at 1 week after photothrombotic occlusion or sham surgery. Brain regions were dissected into infarct, thalamus, and hippocampus, and proteins were extracted by using radioimmunoprecipitation assay buffer. Protein levels of apoptosis, autophagy, neuronal, glial, vascular, and neurodegenerative-related markers were analyzed by western blotting. Our results showed that TTC+ brains display similar relative changes in most of the markers compared with TTC− brains. In addition, we validated that these analyses can be performed in the infarct as well as other brain regions such as the thalamus and hippocampus. Our findings demonstrate that TTC+ brains are reliable for protein analyses using western blotting. Widespread adoption of this approach will be key to lowering the number of animals used while maximizing data.

## Introduction

2,3,5-Triphenyltetrazolium chloride (TTC) is a marker of metabolic function and represents a reliable indicator of ischemic areas in experimental stroke models. TTC is a colorless water-soluble dye that is reduced by the mitochondrial enzyme succinate dehydrogenase of living cells into a water-insoluble, light sensitive compound (formazan) that turns healthy/normal tissue deep red. In contrast, damaged/dead tissue remains white showing the absence of living cells, and thereby indicating the infarct region (Bederson et al., 1986; Li et al., 1997). TTC staining is a widely used technique in stroke and brain injury research because it is simple, cost effective, and offers a rapid visualization of infarct and penumbral areas (Benedek et al., 2006).

Currently, TTC is used as an immersion staining of fresh brain slices (Bederson et al., 1986) or by intracardiac injection (Sun et al., 2012). However, in the vast majority of studies, these TTC-stained brains are immediately discarded after infarct assessment as it is unclear whether the tissue is suitable for subsequent cellular and molecular analyses. Western blotting is a common method used to detect and analyze protein levels. This technique involves the separation of denatured proteins based on their molecular weight and visualization using antibodies specific to the target protein (Burnette, 1981; Liu et al., 2014). The simplicity and relevance of the method has led to its expansive application as a ubiquitous research tool in medical biochemistry.

TTC staining has been shown to be compatible for the analysis of some specific proteins. However, previous studies presented some limitations. A previous study by Sun et al. (2012) showed that *in vivo* TTC-labeling was compatible with immunofluorescence, mRNA, and protein analysis. However, this study only focused on apoptotic markers in penumbral tissue, and therefore it cannot be concluded that this technique can be used to study other markers and other brain regions. In addition, TTC was administered transcardially, which is highly toxic to the animals, instead of immersing the brain sections in a TTC-staining solution. Another study by Kramer et al. (2010) demonstrated that TTC processing *ex vivo* after middle cerebral artery occlusion in rats can be used for quantitative gene and protein expression analyses using RT-PCR and western blot. However, this study only analyzed the protein expression profile of the metalloprotease-disintegrin ADAM12 and the housekeeping protein β-actin. Additional work in this field has also demonstrated that TTC-stained brain sections can also be used for immunohistochemical quantification of Collagen IV and immunofluorescence analyses (Li L. et al., 2018; Li Z. et al., 2018).

Taken into account previous data, the main objective of the present study was to investigate whether TTC-treated tissues can be processed for downstream biochemical analyses, specifically by western blotting. We aimed to rigorously compare the protein expression profile of wide range of commonly used markers between TTC-treated (TTC+) and untreated (TTC−) brains across a number of different brain regions. We hypothesized that TTC treatment would not interfere with protein quantification in the regions studied.

## Materials and Methods

The data that support the findings for this study are available from the corresponding author on reasonable request.

### Experimental Design

Animal research was undertaken in accordance with the ARRIVE guidelines (Animal Research: Reporting of *in vivo* Experiments). Experiments were approved by the University of Newcastle Animal Care and Ethics Committee (A-2013-340) and conducted in accordance with the New South Wales Animals Research Act and the Australian Code of Practice for the use of animals for scientific purposes. A total of 24 mice were randomly allocated to one of four groups: TTC− sham, TTC− stroke, TTC+ sham, and TTC+ stroke. At day 0, mice were subjected to photothrombotic occlusion or sham surgery. At day 7 post-stroke, mice were euthanized. Brains were collected and sliced using a matrix device into 2 mm coronal sections. One cohort (sham *n* = 6; stroke *n* = 6) was stained by TTC and the other cohort (sham *n* = 6; stroke *n* = 6) was not stained.

### Sample Size Calculation

Sample size was estimated using G^*^Power 3.1 software. To determine the sample size required for this study, we used western blot data from our recent study (NeuN; sham = 1.00 ± 0.04 versus stroke = 0.65 ± 0.06; *n* = 7 per group). Allowing a type 1 error of 5%, α = 0.05 with the power of 80%, β = 0.2 we calculated a sample size of six mice per group (Sanchez-Bezanilla et al., 2019).

### Animals

C57BL/6 male mice (10 weeks old) were obtained from the Animal Services Unit at the University of Newcastle. Mice were maintained in a temperature (21°C ± 1) and humidity (∼55%) controlled environment with food and water available *ad libitum*. Lighting was on a 12:12 h reverse light–dark cycle (lights on 19:00 h) with all procedures conducted in the dark phase. In all experiments, mice were acclimatized to the environment for a minimum of 7 days prior to the start of the experiment. Mice were housed between 2 and 4 per cage.

### Photothrombotic Occlusion

Photothrombotic occlusion was performed as previously described (Ong et al., 2017c; Sanchez-Bezanilla et al., 2019). Briefly, mice were anesthetized by 2% isoflurane (Isothesia #029404, Henry Schein) during surgical procedures on a temperature controlled (37°C ± 1) stereotaxic frame. The skull was exposed by incision of the skin along the midline of the scalp. Rose Bengal (200 μl, 10 mg/ml solution in sterile saline, Sigma–Aldrich #330000, United States) was injected intraperitoneally. After 8 min, the skull was illuminated for 15 min by a 4.5 mm diameter cold light source (Zeiss KL2500LCD, Germany) positioned at 2.2 mm left lateral of bregma 0.0 mm, targeting the left motor and somatosensory cortices. For the sham group, the same surgical procedure was conducted except Rose Bengal was replaced with 200 μl of sterile saline (0.9% NaCl, Pfizer #SYM273384, Australia).

### Brain Regions Preparation and Collection

**FIGURE 1:**
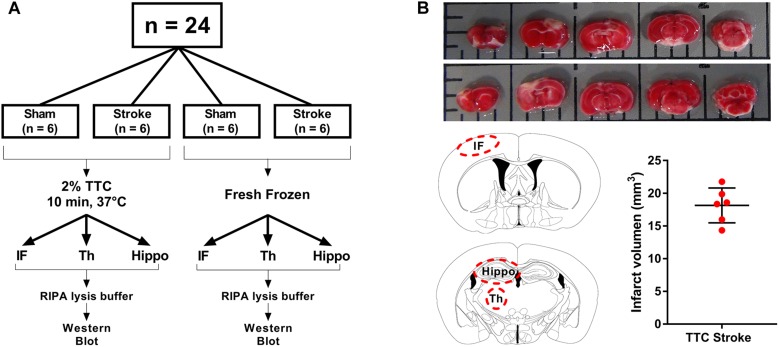
Schematic illustration of the experimental design. **(A)** Mice were randomly allocated to sham or stroke group. At day 7 post-stroke mice were euthanized and brains were sliced into 2 mm thin sections using a matrix device. Brain samples were divided into two different groups. The brain slides from the first group (TTC+) were stained with 2% TTC at 37°C for 10 min, then further dissected into infarct (IF), thalamus (Th), and hippocampus (Hippo) and frozen at −80°C. The slices from the second group (TTC−) were dissected and immediately frozen at −80°C. **(B)** Top panel: images of TTC+ mouse brain slices after photothrombotic stroke. After the brain have been sliced, we placed the 2 mm section on a grid mat. Once we have taken a picture of one side (rostral, row 1) of the brain, we flipped the sections and take a picture of the other side (caudal, row 2). Bottom panel: schematic illustration showing the location of the examined regions (IF, Th, and Hippo) (left). Quantification of the IF volume in TTC+ stroke mice (right).

At day 7, mice were deeply anesthetized via intraperitoneal injection of 200 μl of sodium pentobarbital (Lethabarb #3729556, Virbac, Australia). Mice were transcardially perfused with ice cold 0.9% NaCl (UNIVAR #AJA465-2.5KG) with 0.1% diethylpyrocarbonate (Sigma–Aldrich #D5758, Australia) for 3 min. Brains were dissected and sliced using a matrix device (Zivic Instruments #5325, United States) into 2 mm coronal sections from the olfactory bulb to the cerebellum. For TTC+ brains (sham *n* = 6, stroke *n* = 6), sections were taken and incubated in 2% TTC (Sigma–Aldrich #T8877) in saline at 37°C for 10 min. TTC+ brain sections were captured with a digital camera (Sony HDR-PJ790) for infarct volume analyses. Infarct (identified as non-TTC-stained region in stroke mice and equivalent region in sham mice), thalamus (Bregma −1.0 to −2.2), and hippocampus (Bregma −1.5 to −2.5) areas were identified and dissected from the coronal sections and then frozen at −80°C. For TTC− brains (sham *n* = 6, stroke *n* = 6), same brain regions were dissected (infarct identified as part of the cortex that appeared to be occluded) and immediately frozen at −80°C (Figure 1A).

### Infarct Volume Quantification

Contralateral and ipsilateral hemisphere and infarct areas were traced using ImageJ software. Infarct volume was corrected for edema using the formula: corrected infarct volume (mm^3^) = infarct volume × (contralateral volume/ipsilateral volume). Edema was calculated by infarct volume minus corrected infarct volume (Murtha et al., 2016; Figure 1B).

### Protein Extraction and Western Blotting

Western blot was performed as previously described with minor modification (Ong et al., 2017b, 2018). Infarct, thalamus, and hippocampus samples were sonicated using a UP50H microsonicator (Hielscher Ultrasonics GmbH, Germany) for 3 × 30 s pulses at 4°C in radioimmunoprecipitation assay (RIPA) buffer [25 mM Tris buffer pH 7.4, 150 mM NaCl, 0.5% sodium deoxycholate, 0.1% SDS, 1% Triton X-100, 1 protease inhibitor cocktail tablet (every 50 ml), 1× phosphatase inhibitor cocktail tablet (every 50 ml)]. Samples were centrifuged at 14,000 ×*g* for 20 min at 4°C. The supernatant fractions were collected. Protein concentrations were determined using a Pierce BCA protein assay kit (Thermo Fisher Scientific #23225, United States) according to the manufacturer’s instructions. Each sample’s final concentration was adjusted to 1.5 mg/ml and aliquoted. Samples were mixed with sample buffer (2% SDS, 10% glycerol, 1% dithiothreitol). Equal amounts of tissue protein samples were electrophoresed into Bio-Rad Criterion TGC stain-free 4–20% gels (Bio-Rad #5678095, Australia). To ensure that an equal amount of protein was loaded, one gel per region was scanned using ChemiDoc XRS+ system (Bio-Rad) before the transfer (Supplementary Figure S1). Gels were transferred to polyvinylidene difluoride membranes (Bio-Rad #1620177, Australia). PVDF membranes were washed three times in Tris-buffered saline with Tween (TBST) (150 mM NaCl, 10 mM Tris, 0.075% Tween-20, pH 7.5) and incubated in 5% skim milk powder in TBST for 1 h at room temperature. Membranes were incubated with primary antibodies (amyloid-β, α-synuclein, NeuN, glial fibrillary acidic protein (GFAP), ADLH1L1, PSD95, Tau5, P-Tau, P-Tau Ser396, synaptophysin, LC3, caspase-3, CD11b, VEGFA, Collagen IV, and CD31) overnight at 4°C and secondary antibody for 1 h at room temperature (see Table 1 for antibodies details and concentration). In between each incubation step, membranes were washed three times in TBST. Membranes were visualized on an Amersham Imager 600 using Luminata Classico (Millipore #WBLUC0500, Australia) or Luminata Forte (Millipore #WBLUF0500, Australia) western blotting detection reagents. The density of the bands was measured using Amersham Imager 600 analysis software. The housekeeping protein β-actin was used as a loading control to normalize the levels of target protein. For representative images, two out of six bands per group were cropped from the raw blots and aligned together. The bands were then minimally processed in term of brightness and contrast, and presented as the representative blots in the figures.

**TABLE 1:** List of antibodies used for western blotting.

	**Target**	**Description**	**Sources of antibodies**	**Dilution**
Apoptosis and autophagy	Caspase-3	Caspase-3 is an intracellular proteases that mediates cell death and plays a critical role in apoptosis. Activation of caspase-3 requires proteolytic processing (Fernandes-Alnemri et al., 1994).	Sigma–Aldrich, anti-Caspase 3, #C8487-200UL	1:1000
	LC3	Light chain 3 (LC3) is an autophagy marker. Cleavage of LC3 yields the cytosolic LC3-I form. During autophagy, LC3-I is converted to LC3-II permitting LC3 to become associated with autophagic vesicles (Kabeya et al., 2000).	Sigma–Aldrich, anti-LC3, #L8918-200UL	1:1000
Neuronal	NeuN	Neuronal nuclei (NeuN) is a nuclear protein expressed in most post-mitotic neurons of the central and peripheral nervous systems (Mullen et al., 1992). Marker for mature neurons.	Cell Signaling, anti-NeuN (D3S31), #12943	1:2000
	Synaptophysin	Synaptophysin is an integral membrane protein of small synaptic vesicles in the brain (Wiedenmann et al., 1986).	Millipore, anti-Synaptophysin, #MAB329	1:10,000
	PSD95	Postsynaptic density protein 95 (PSD95) is a scaffolding protein involved in the assembly and function of the postsynaptic density complex (Chetkovich et al., 2002).	Cell Signaling, anti-Postsynaptic density protein 95, #2507	1:1000
Glial	GFAP	Glial fibrillary acidic protein (GFAP) forms intermediate filaments in astroglial cells and modulate their motility and shape. GFAP filaments are characteristic of differentiated and mature brain astrocytes (Eng et al., 2000).	Cell Signaling, anti-Glial Fibrillary Acidic Protein (GA5), #3670	1:5000
	ALDH1L1	10-Formyltetrahydrofolate dehydrogenase (ALDH1L1) is a multidomain protein that serves as a CNS astrocyte marker (Cahoy et al., 2008).	Millipore, anti-ALDH1L1 (N103/39), #MABN495	1:2000
	CD11b	Cluster of differentiation molecule 11b (CD11b) is a transmembrane protein expressed by neutrophils, monocytes, macrophages, and microglia (Solovjov et al., 2005).	Abcam, anti-CD11b, #ab75476	1:2000
Vascular	VEGFA	Growth factor active in angiogenesis, vasculogenesis, and endothelial cell growth. Induces endothelial cell proliferation, promotes cell migration, inhibits apoptosis, and induces permeabilization of blood vessels (Leung et al., 1989).	Abcam, anti-VEGFA #ab46154	1:1000
	CD31 (PECAM-1)	CD31 (Platelet Endothelial Cell Adhesion Molecule-1, PECAM-1) is a cell adhesion molecules expressed by circulating platelets, monocytes, neutrophils, some T cells, and makes up a large portion of endothelial cell intercellular junctions. Modulates cell adhesion, endothelial cell migration, and angiogenesis (Newman, 1997).	Cell Signaling, anti-CD31 (PECAM-1) (D8V9E) #77699 Sigma–Aldrich, anti-PECAM-1 #SAB4502167	1:1000 1:1000
	Collagen IV	Type IV collagen is the major structural component of basement membranes. Type IV collagen is a network-forming collagen that provides a molecular scaffold and interacts with cells, growth factors, and other basement membrane components such as laminin, nidogen, and perlecan (Kuhn, 1995).	Abcam, anti-Collagen IV #ab6586	1:1000
Neurodegeneration	Amyloid-β	Amyloid-β (Aβ) peptide produced through sequential proteolytic processing of amyloid precursor protein, and it is prone to aggregate in pathological conditions (Haass and Selkoe, 2007; Walsh and Selkoe, 2007).	Biolegend, anti-Amyloid-β (6E10), # SIG-39320	1:1000
	α-Synuclein	α-Synuclein (α-Syn) is expressed in brain, primarily in presynaptic nerve terminals. Although the exact function has not been determined, it has been linked to the prominent neurodegenerative disorders (Maroteaux et al., 1988; van der Putten et al., 2000).	BD Bioscience, anti-α-Synuclein, #610787	1:1000
	Tau5	Tau5 detects total levels of Tau. Tau are a microtubule-associated proteins that bind to the tubulin subunits of microtubule structures, and promote and stabilize microtubule assembly (Avila et al., 2004; Johnson and Stoothoff, 2004).	Millipore, anti-Tau5, #MAB361	1:2000
	P-Tau (Ser400/ Thr403/Ser404)	Phospho-Tau (P-Tau) recognizes endogenous levels of tau protein when phosphorylated at Ser400 or Thr403 or Ser404. Studies have shown that tau phosphorylation at Ser404 destabilizes microtubules and that tau is hyperphosphorylated at Ser404 in Alzheimer’s disease (Evans et al., 2000). Phosphorylation decreases the ability of tau to bind to microtubules, destabilizing the structure and driving it toward disassembly. Neurofibrillary tangles are a major hallmark of Alzheimer’s disease; these tangles are composed of hyperphosphorylated tau (Johnson and Stoothoff, 2004).	Cell Signaling, anti-Phospo-Tau (Ser400/Thr403/Ser404), #11837	1:1000
	P-Tau (Ser396)	Phospho-Tau (Ser396) (P-Tau Ser396) detects endogenous levels of Tau only when phosphorylated at serine 396. Phosphorylation at Ser396 has shown to destabilize microtubules and contribute to different neurological disorders (Bramblett et al., 1993; Evans et al., 2000).	Cell Signaling, anti-Phospo-Tau (Ser396), #9632	1:1000
Housekeeping	β-Actin	β-Actin is a cytoskeletal housekeeping protein.	Sigma–Aldrich, monoclonal anti-β-actin-HRP antibody, A3854	1:50,000
Secondary	Rabbit IgG	Secondary antibody.	Bio-Rad, Anti-Rabbit-HRP antibody, #170-6515	1:7500
	Mouse IgG	Secondary antibody.	Bio-Rad, Anti-Mouse-HRP antibody, #170-6516	1:10,000

### Statistics

All data were expressed as a fold increase of the mean ± SEM for each group relative to the mean of the TTC− sham group. These data were analyzed by using GraphPad Prism v7.02. Data from western blotting were analyzed using two-way analysis of variance (ANOVA) followed by Sidak’s *post hoc* comparison. The significant differences shown on the graphs with asterisks (^*^) refer to the *post hoc* tests. All differences were considered to be significant at *p* < 0.05.

## Results

### Apoptotic and Autophagy Markers

**FIGURE 2:**
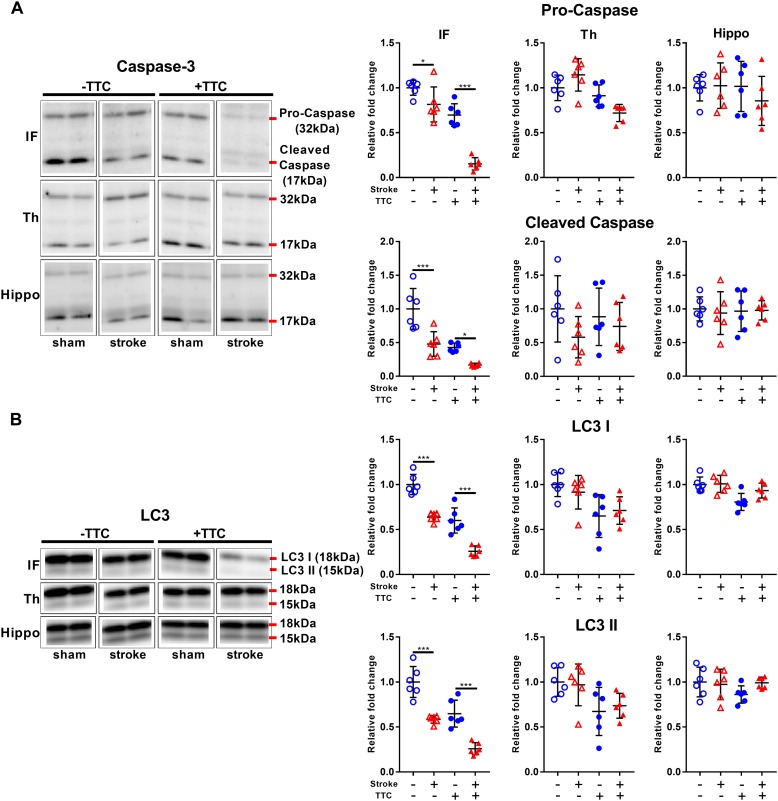
Apoptotic and autophagy markers. Representative western blot and quantification of caspase-3 (pro-caspase and cleaved caspase) **(A)** and LC3 (LC3I and LC3II) **(B)** within the infarct (IF), thalamus (Th), and hippocampus (Hippo). Mean ± SEM (two-way ANOVA and Sidak’s multiple comparisons). 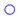
TTC– sham; 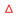
TTC– stroke; 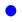
TTC+ sham; 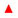
TTC+ stroke. ^*^p < 0.05, ^∗∗∗^*p* < 0.001.

Protein levels of pro-caspase-3 and cleaved caspase-3 were significantly reduced in stroke mice compared to sham in the infarct area of both the TTC− (pro-caspase-3 *p* = 0.0419; cleaved caspase-3 *p* = 0.0001) and the TTC+ (pro-caspase-3 *p* < 0.0001; cleaved caspase-3 *p* = 0.0432) group. We found no significant differences in neither the thalamus nor hippocampus (Figure 2A). Quantification of LC3 showed a significant decrease in stroke mice compared to sham in the infarct of the TTC− (LC3I *p* < 0.0001; LC3II *p* < 0.0001) and TTC+ (LC3I *p* < 0.0001; LC3II *p* < 0.0001) group. Changes in the protein expression levels in the thalamus and hippocampus were not statistically significant between sham and stroke group (Figure 2B).

### Neuronal Markers

**FIGURE 3:**
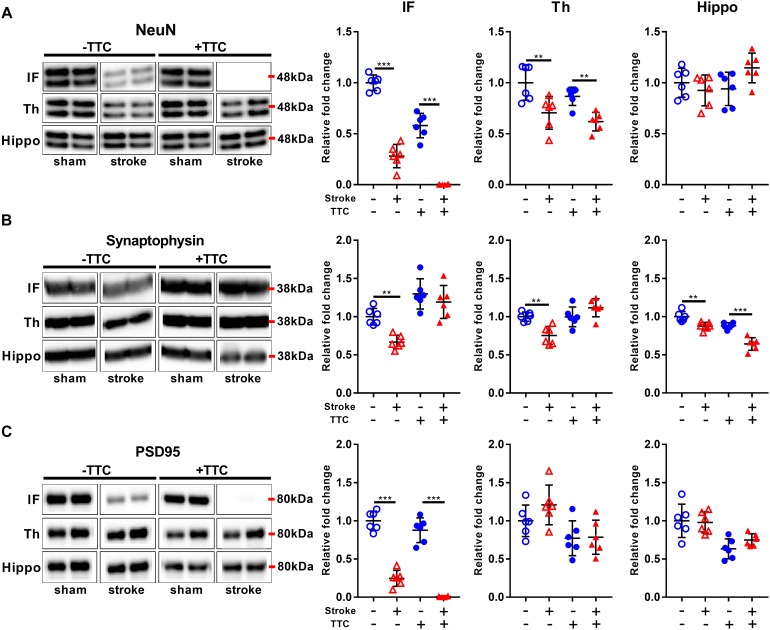
Neuronal markers. Representative western blot and quantification of NeuN **(A)** Synaptophysin **(B)**, and PSD95 **(C)** within the infarct (IF), thalamus (Th), and hippocampus (Hippo). Mean ± SEM (two-way ANOVA and Sidak’s multiple comparisons). 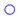
TTC– sham; 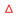
TTC– stroke; 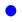
TTC+ sham; 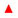
TTC+ stroke. ^∗∗^*p* < 0.01, ^∗∗∗^*p* < 0.001.

Protein levels of NeuN were significantly reduced in stroke mice in the infarct and thalamus areas of both TTC− (NeuN infarct *p* < 0.0001, NeuN thalamus *p* = 0.0021) and TTC+ (NeuN infarct *p* < 0.0001; NeuN thalamus *p* = 0.0086) group. However, no significant differences were found in the hippocampus (Figure 3A). Protein levels of synaptophysin were significantly reduced in the infarct (*p* = 0.0040), thalamus (*p* = 0.0019), and hippocampus (*p* = 0.0042) of TTC− stroke. In the TTC+ group, synaptophysin was significantly reduced just in the hippocampus (*p* < 0.0001) (Figure 3B). We observed a significant decrease in PSD95 in the infarct area of TTC− (*p* < 0.0001) and TTC+ (*p* < 0.0001) brains. However, no significant changes were observed in the other regions (Figure 3C).

### Glial Markers

**FIGURE 4:**
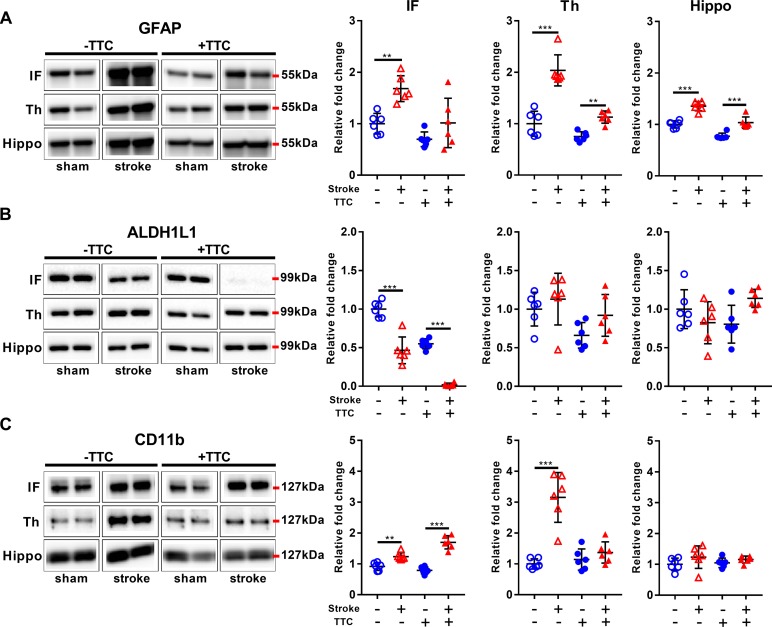
Glial markers. Representative western blot and quantification of GFAP **(A)**, ALDH1L1 **(B)**, and CD11b **(C)** within the infarct (IF), thalamus (Th), and hippocampus (Hippo). Mean ± SEM (two-way ANOVA and Sidak’s multiple comparisons). 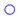
TTC− sham; 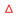
TTC– stroke; 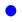
TTC+ sham; 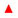
TTC+ stroke. ^∗∗^*p* < 0.01, ^∗∗∗^*p* < 0.001.

Glial fibrillary acidic protein was significantly increased in all investigated regions in the TTC− stroke mice compared to sham (infarct *p* = 0.0015; thalamus *p* < 0.0001; hippocampus *p* < 0.0001). In the TTC+ stroke mice, GFAP was increased in the thalamus (*p* = 0.0100) and hippocampus (*p* < 0.0001) (Figure 4A). In contrast, ALDH1L1 was significantly downregulated in the infarct area of both TTC− (*p* < 0.0001) and TTC+ (*p* < 0.0001) stroke mice (Figure 4B). No significant differences were found in other areas. CD11b showed increased protein levels in the infarct area of TTC− (*p* = 0.0029) and TTC+ (*p* < 0.0001) stroke mice (Figure 4C). CD11b was also increased in the thalamus of TTC− stroke mice (*p* < 0.0001). There were no significant differences in the hippocampus.

### Vascular Markers

We analyzed the expression of Collagen IV, CD31, and VEGFA. VEGFA showed a significant decrease in the infarct area of TTC+ stroked mice. There were no differences in the thalamus or hippocampus (Supplementary Figure S2). However, we could not detect any bands for Collagen IV or CD31. This could be due to the protein extraction protocol was not suitable for these target proteins or the recommended conditions of the antibodies used were not optimized. It will be of considerable interest in future studies, using appropriate lysis buffer and protein extraction procedure as well as validated primary antibodies, to examine vascular markers in TTC-treated tissues.

### Neurodegeneration-Related Markers

**FIGURE 5:**
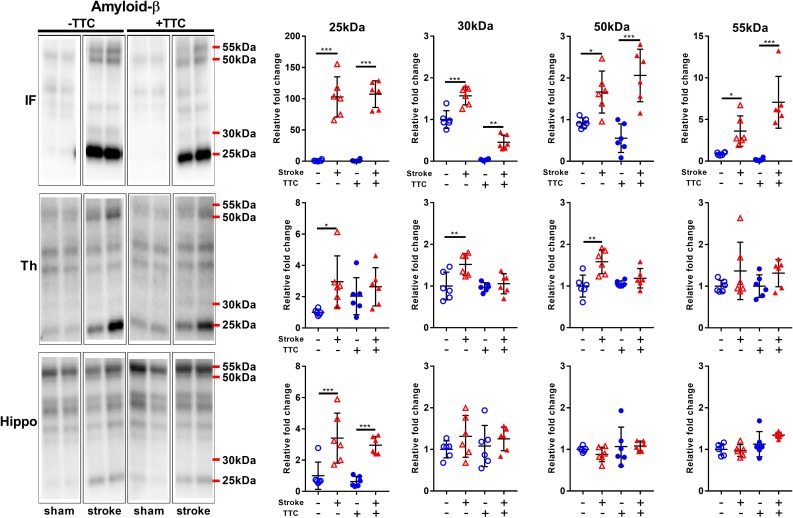
Representative western blot and quantification of amyloid-β expression profile within the infarct (IF), thalamus (Th), and hippocampus (Hippo). Our analyses focused on different molecular weight oligomers (25, 30, 50, and 55 kDa). Mean ± SEM (two-way ANOVA and Sidak’s multiple comparisons). 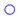
TTC– sham; 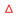
TTC– stroke; 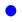
TTC+ sham; 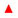
TTC+ stroke. ^*^p < 0.05, ^∗∗^*p* < 0.01, ^∗∗∗^*p* < 0.001.

We analyzed the expression levels of three commonly investigated neurotoxic protein markers. First, we analyzed amyloid-β (Aβ) oligomerization after stroke. Specifically, we quantitated the pentamer (25 kDa), intermediate size oligomers (30 kDa), decamer (50 kDa), and dodecamer (55 kDa). We observed showed a significant increase of all Aβ oligomers studied in the infarct area of both TTC− (pentamer *p* < 0.0001; intermediate size oligomer *p* < 0.0001; decamer *p* = 0.0175; dodecamer *p* = 0.0336) and TTC+ (pentamer *p* < 0.0001; intermediate size oligomer *p* = 0.0047; decamer *p* < 0.0001; dodecamer *p* < 0.0001) stroke mice. In the thalamus, we observed a significant increase in the pentamer (*p* = 0.0366), intermediate size oligomers (*p* = 0.0021), and decamer (*p* = 0.0022) in the TTC− stroke group. Protein levels in the TTC+ stroke group showed a trend toward an increase however was not statistically different to sham animals. Additionally, in the hippocampus we observed a significant increase in the pentamer in both TTC− (*p* = 0.0007) and TTC+ (*p* = 0.0009) group (Figure 5).

**FIGURE 6:**
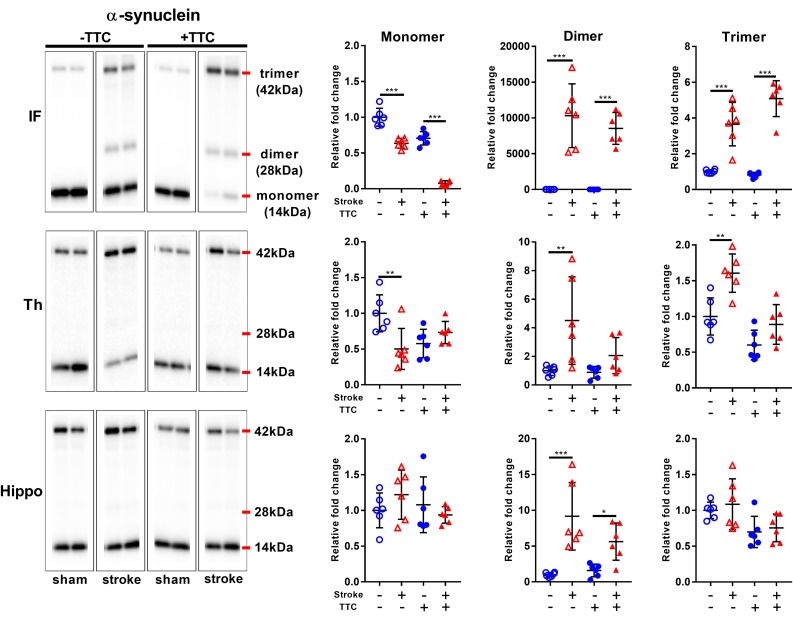
Representative western blot and quantification of α-synuclein expression profile within the infarct (IF), thalamus (Th), and hippocampus (Hippo). Our analysis focused on the monomer (14 kDa), dimer (28 kDa), and trimer (42 kDa). Mean ± SEM (two-way ANOVA and Sidak’s multiple comparisons). 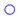
TTC– sham; 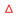
TTC– stroke; 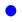
TTC+ sham; 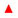
TTC+ Stroke. ^*^p < 0.05, ^∗∗^*p* < 0.01, ^∗∗∗^*p* < 0.001.

Secondly, we evaluated the monomer (14 kDa), dimer (28 kDa), and trimer (42 kDa) levels of α-Syn. In the infarct, we found a significant decrease of monomer levels, and a corresponding significant increase in α-Syn dimer and trimer levels in both TTC− (monomer *p* < 0.0001; dimer *p* < 0.0001; trimer *p* < 0.0001) and TTC+ (monomer *p* < 0.0001; dimer *p* < 0.0001; trimer *p* < 0.0001) group. In the thalamus, a significant reduction of monomer levels, and a corresponding significant increase in α-Syn trimer levels were observed in the TTC− group only (monomer *p* = 0.0026; dimer *p* = 0.0033; trimer *p* = 0.0011). The TTC+ group showed no significant differences in this region. In the hippocampus, we found no statistically significant changes in the monomer or trimer, but we observed a significant increase in the dimer levels in both groups (dimer TTC− *p* < 0.0001; dimer TTC+ *p* = 0.0378) (Figure 6).

**FIGURE 7:**
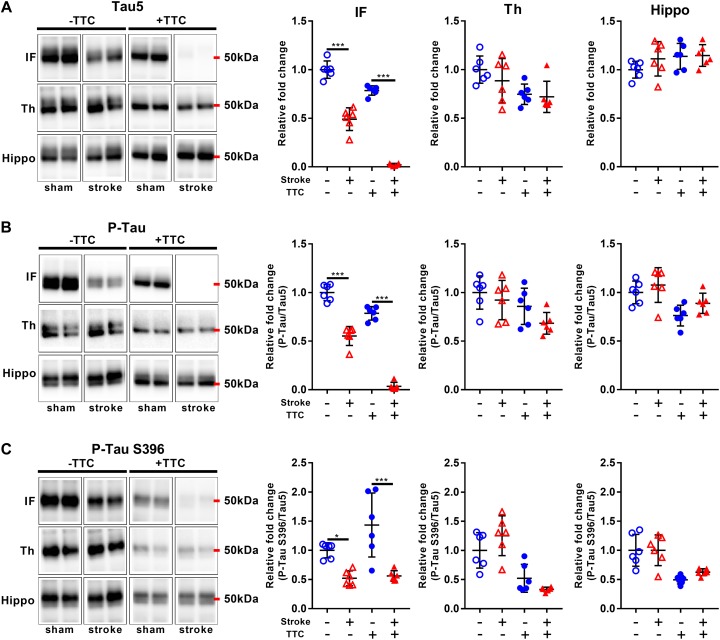
Representative western blot and quantification of Tau5 **(A)**, P-Tau **(B)**, and P-Tau S396 **(C)** within the infarct (IF), thalamus (Th), and hippocampus (Hippo). Mean ± SEM (two-way ANOVA and Sidak’s multiple comparisons). 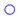
TTC– sham; 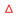
TTC– stroke; 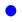
TTC+ sham; 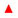
TTC+ stroke. ^*^p < 0.05, ^∗∗∗^*p* < 0.001.

Lastly, we evaluated protein levels of total Tau and two site-specific phosphorylated forms, which have been associated with the formation of neurofibrillary tangles in neurodegenerative diseases. The levels of total Tau, as evaluated by Tau5, were decreased in the infarct area of TTC− (*p* < 0.0001) and TTC+ (*p* < 0.0001) group. Thalamus and hippocampus showed no changes. We then assessed the levels of Phospho-Tau (Ser400/Thr403/Ser404) and Phospho-Tau Ser396 relative to the total Tau. Reduced P-Tau and P-Tau (Ser396) levels were observed in the infarct after stroke in both group (P-Tau TTC− *p* < 0.0001; P-Tau Ser396 TTC− *p* = 0.0203; P-Tau TTC+ *p* < 0.0001; P-Tau Ser396 TTC+ *p* < 0.0001). No significant differences were found in the thalamus and hippocampus (Figure 7).

## Discussion

In this study, we investigated whether TTC+ tissues can be processed for downstream biochemical analyses, specifically by western blotting. Our results suggest that brain samples that are processed by TTC staining can be routinely used for protein analyses. Overall, the hippocampus shows same relative changes between TTC− and TTC+ group for all the markers studied, whereas thalamus and infarct differ in a couple of makers (summary of the results Supplementary Figure S3). This study demonstrates and further supports the usage of TTC staining for downstream protein analysis.

Previous studies have demonstrated that TTC+ stroke brains can be used for histology, gene, and protein expression analyses (Kramer et al., 2010; Sun et al., 2012; Li L. et al., 2018; Li Z. et al., 2018). Taking all these studies together suggest that TTC-stained tissue can be used for biochemical analyses without restrictions. However, there are a few limitations regarding the protein expression analyses that we wanted to address in our study. Firstly, previous studies have just focused on a limited number of markers, mainly apoptotic markers and housekeeping proteins (Kramer et al., 2010; Sun et al., 2012; Mohammad-Gharibani et al., 2014; Gharibani et al., 2015; She et al., 2018) and therefore it cannot be definitely asserted that other proteins can also be studied after TTC treatment. Secondly, other studies did not study the effects of TTC on protein modification such as phosphorylation and aggregation. Finally, the penumbra and surrounding areas have been the primary focus; however, there may be different reactions to TTC in other brain regions. Therefore, here we analyzed a total of 16 markers, which include apoptotic, autophagy, neuronal, glial, vascular, and neurodegeneration-related markers. In addition, we analyzed the changes in the primary infarct area and in regions that have been previously linked with secondary neurodegeneration processes (thalamus and hippocampus) (Xie et al., 2011; Ong et al., 2017a; Baumgartner et al., 2018; Sanchez-Bezanilla et al., 2019).

We observed that in the infarct region all the markers with the exception of synaptophysin, VEGFA, and GFAP presented the same relative changes in the TTC− and TTC+ group. In the thalamus, TTC-stained and non-stained tissue showed similar relative changes in 9 out of 13 markers studied. Aβ, α-Syn, CD11b, and synaptophysin were the only markers that exhibited different results when comparing TTC+ and TTC− in the thalamus. In the hippocampus, all the markers showed the same changes in both group. The discrepancies observed in the infarct region can be explained by the procedure used to define the area considered infarct. While in the TTC+ group the infarct area is clearly delineated as the colorless area, in the TTC− group is not easy to visually determine the infarct area. Therefore, surrounding tissue could have been potentially collected as part of the infarct in the TTC− group. Further, the differences observed in some of the markers studied could be related to the TTC staining protocol. Firstly, proteolysis may occur for some proteins when the brain sections are incubated at 37°C for 10 min. Secondly, the formazan pigment (deep red color) may interfere with the protein estimation using the BCA colorimetric protein assay. Interestingly, we observed that Tau5, P-Tau (Ser400/Thr403/Ser404), and P-Tau (S396) showed the same relative changes in all the areas, suggesting that TTC staining does not present a major interference with protein phosphorylation. We also observed that TTC does not interfere with protein aggregation processes in the infarct and hippocampus as both Aβ and α-Syn presented the same relative changes. From these results, we can conclude that brain sections that are processed by TTC staining are viable for western blotting. While the usage of western blotting following TTC staining can produce high-throughput qualitative and semi-quantitative data for a variety of protein markers, the end-user should optimize the protocols and conditions accordingly.

In this study, we provide a useful procedure to repurpose brain sections after they have been treated with TTC staining, which brings multiple advantages. Firstly, TTC staining is a simple, rapid, and inexpensive method that allows to delineate the size of the cerebral infarct area and distinguish from the penumbra and intact tissue. Therefore, we can precisely select the area of the brain that it is more suited for protein expression analyses. Furthermore, we can select brains with the same infarct size and reduce the experimental variability. This procedure can also be used to investigate protein modification such as protein phosphorylation and aggregation. Another important benefit of this approach is that it permits a reduction in the number of animals required for investigation as the same brains can be used for infarct quantification and protein quantification. Specifically, we demonstrated that we could use western blotting for the simultaneous assessment of a large number of different markers as well as protein phosphorylation and aggregation after brains have been treated with TTC. In conclusion, our study demonstrates that TTC brains should not be discarded as they can be used for further protein analyses of a wide variety of commonly used markers.

## Data Availability

The datasets generated for this study are available on request to the corresponding author.

## Ethics Statement

The animal study was reviewed and approved by the University of Newcastle Animal Care and Ethics Committee (A-2013-340).

## Author Contributions

SS-B and LKO designed the research, performed the experiments, and prepared the first draft of the manuscript. FRW and MN supervised the study. SS-B and LKO analyzed the data and interpreted the results. All authors reviewed and contributed to the final version of the manuscript.

## Conflict of Interest Statement

The authors declare that the research was conducted in the absence of any commercial or financial relationships that could be construed as a potential conflict of interest.
