# Supplementary material for: Can We Use 2,3,5-Triphenyltetrazolium Chloride-Stained Brain Slices for Other Purposes? The Application of Western Blotting
Source: Front Mol Neurosci. 2019 Jul 30;12:181. doi: 10.3389/fnmol.2019.00181 (PMC6682641; doi:10.3389/fnmol.2019.00181)
Supplement: Supplementary file 1 [file Table_1.DOCX]

**Supplementary material**

**Supplementary Figure S1**

**
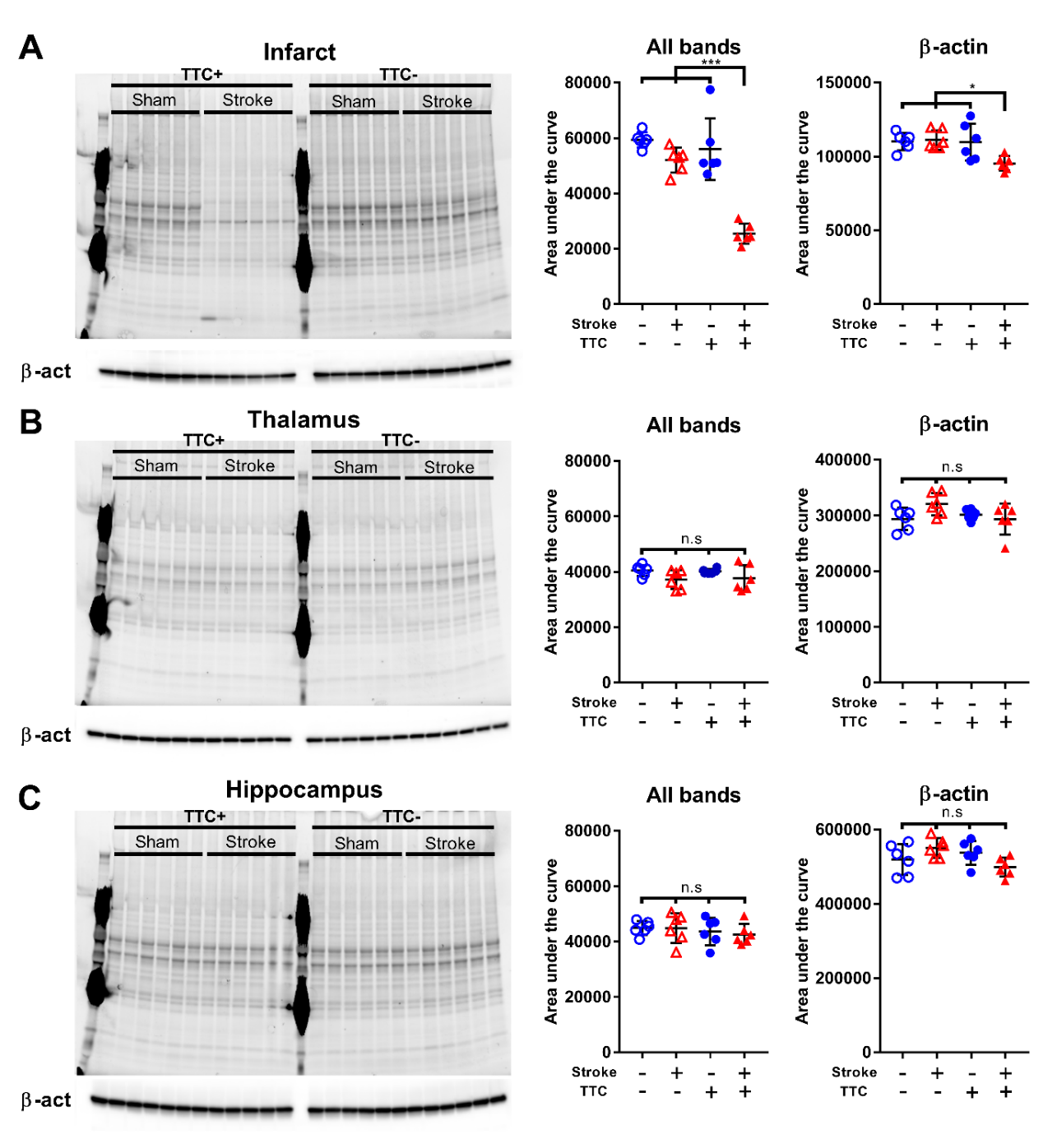
**

**Supplementary Figure S1.** Images of the total amount of proteins loaded in the gels and β-actin (β-act) representative gels. To ensure that equal amount of protein was loaded, one gel per region was scanned using ChemiDoc XRS+ system before the transfer. The housekeeping protein β-actin was also used as a loading control to normalised the levels of protein detected. (A) Stroke brains treated with TTC (TTC+) present a significantly lower amount of total protein as well as less β-actin in the infarct area. This might explained due to the lack of living tissue in the infarct area of TTC+ stroke group. In the TTC- stroke group surrounding tissue could have been potentially collected together with the infarct, increasing the amount of total protein. We could not observed any significant differences in the total protein loaded or β-actin levels in the thalamus (B) or hippocampus (C). Mean ± SEM (two-way ANOVA and Sidak’s multiple comparisons). **○**TTC- Sham; △TTC- Stroke; **●**TTC+ Sham; ▲TTC+ Stroke. * p < 0.05, *** p < 0.001, n.s = not significant

**Supplementary Figure S2**

**
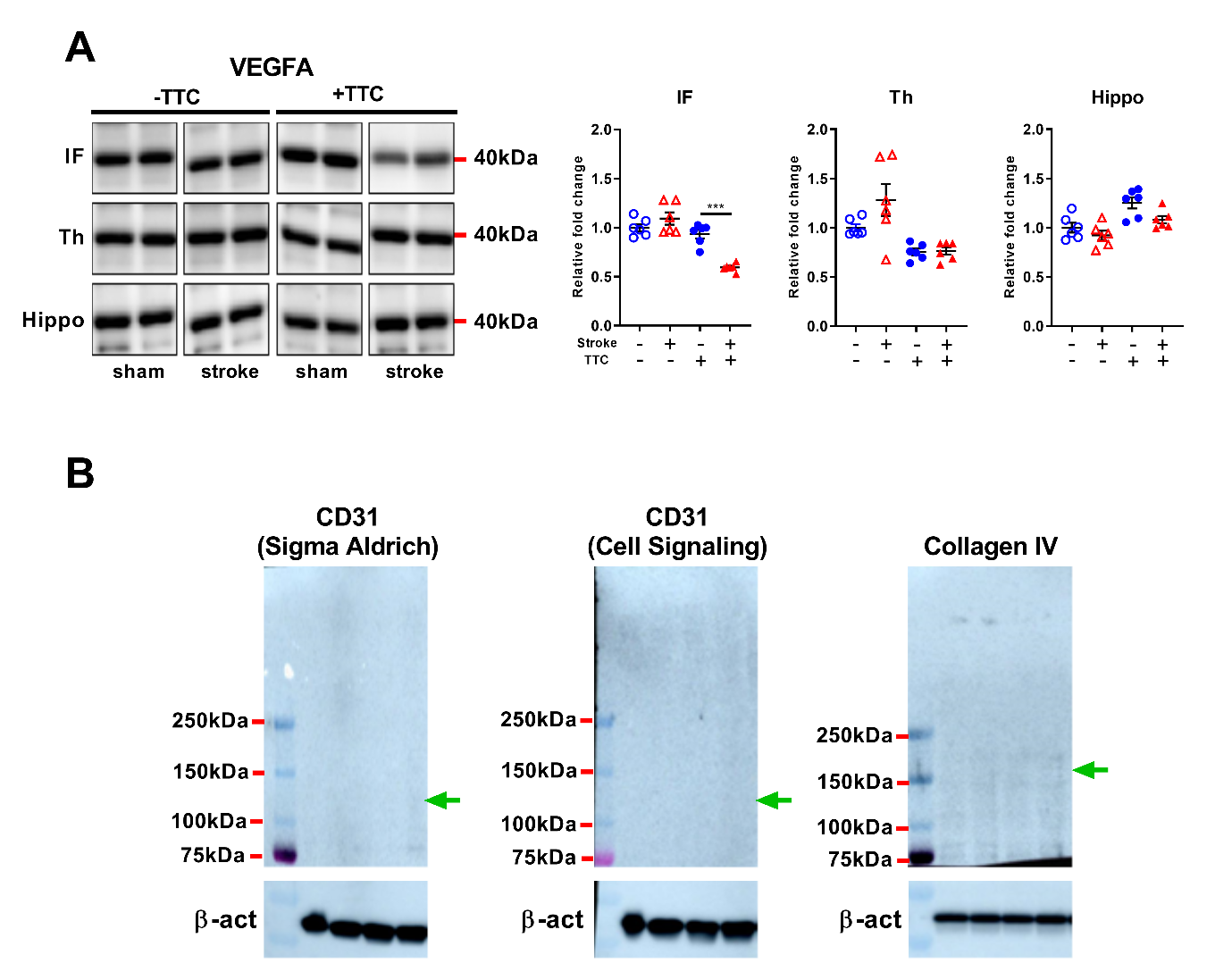
**

**Supplementary Figure S2.** Vascular markers. **(A)** Representative western blot and quantification of VEGFA within the infarct (IF), thalamus (Th) and hippocampus (Hippo). **(B)** Western blot of CD31 (expected molecular weight: 130kDa) and collagen IV (expected molecular weight: 160-190kDa). No bands could be detected. The loading controls were performed by the analysis of β-actin (β-act). Green arrows show expected molecular weight. Mean ± SEM (two-way ANOVA and Sidak’s multiple comparisons). **○**TTC- Sham; △TTC- Stroke; **●**TTC+ Sham; ▲TTC+ Stroke. *** p < 0.001.

**Supplementary Figure S3**

**
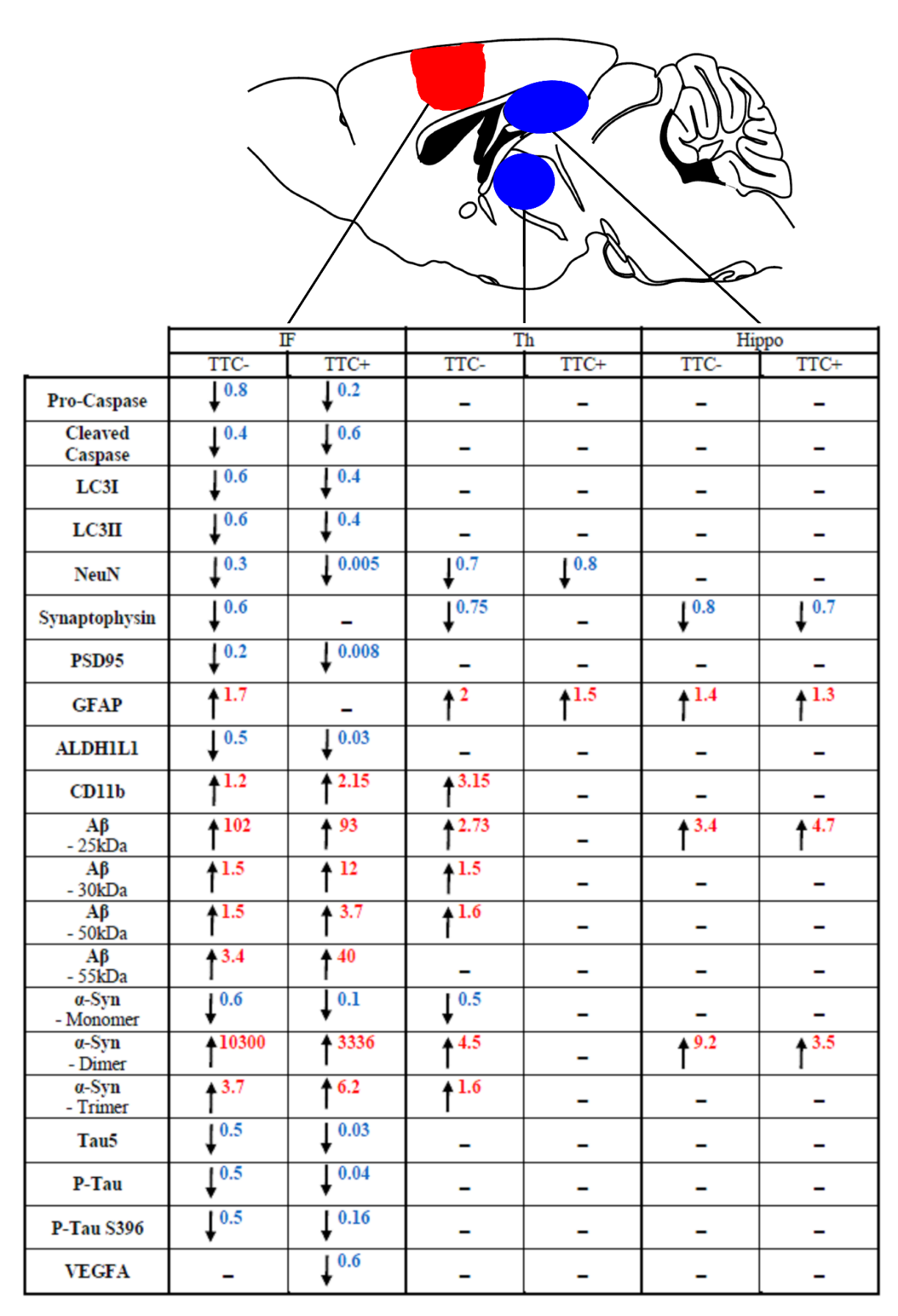
**

**Supplementary Figure S3.** A summary of the changes observed in the infarct (IF), thalamus (Th) and hippocampus (Hippo) evoked by stroke when brains are TTC-stained (TTC+) and non-TTC-stained (TTC-). All changes are presented as fold change of stroke group relative to sham. Decreases in protein expression in stroke mice relative to sham are shown by downward arrows and increases by upward arrows. No changes are indicated by a horizontal dash.
